# 221. Performance of risk scores in predicting infective endocarditis Staphylococcus aureus bacteremia in a prospective Asian cohort

**DOI:** 10.1093/ofid/ofad500.294

**Published:** 2023-11-27

**Authors:** Jinghao Nicholas Ngiam, Matthew CY Koh, Wilson GW Goh, Sai Meng Tham, Sophia Archuleta, Dale Fisher, Louis Chai, Paul Tambyah

**Affiliations:** National University Health System, Singapore, Singapore; National University Health System, Singapore, Singapore; NUHS, Singapore, Not Applicable, Singapore; NUHS, Singapore, Not Applicable, Singapore; NUHS, Singapore, Not Applicable, Singapore; National University Health System Singapore, Singapore, Not Applicable, Singapore; NUHS, Singapore, Not Applicable, Singapore; National University Hospital, Singapore, Singapore, Not Applicable, Singapore

## Abstract

**Background:**

Several risk scores have been derived to predict the risk of infective endocarditis (IE) amongst patients with *Staphylococcus aureus* bacteraemia (SAB). This helps to guide clinical management, including the need for transoesophageal echocardiography (TEE).

**Methods:**

We prospectively studied 634 patients admitted with SAB. Baseline clinical characteristics and laboratory results recorded, and patients were followed up prospectively. The cohort was stratified into those with or without IE, and the PREDICT Day 1, Day 5 and VIRSTA scores were tabulated. Area under receiver operating characteristic (AUC) curves were constructed to compare the performance of each score.

**Figure:**
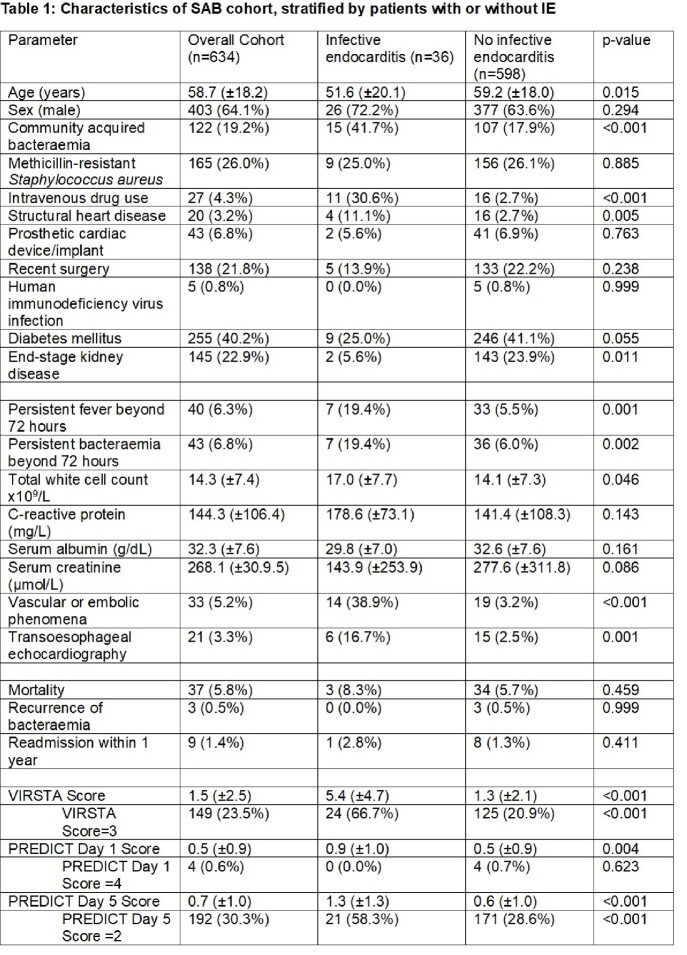

**Results:**

Of the 634 patients examined, 36 (5.7%) had IE. These patients were slightly younger (51.6±20.1 vs 59.2±18.0 years, p=0.015), but tended to have community acquisition of bacteraemia (41.7% vs 17.9%, p< 0.001), intravenous drug use (30.6% vs 2.7%, p< 0.001), pre-existing structural heart disease (11.1% vs 2.7%, p=0.005), and persistent bacteraemia beyond 72 hours (19.4% vs 6.0%, p=0.002). There was no significant difference in mortality (8.3% vs 5.7%, p=0.459). The VIRSTA score had the best performance in predicting IE (AUC 0.76, 95%CI 0.66-0.86) compared with PREDICT Day 1 and Day 5. A VIRSTA score of < 3 had the best negative predictive value (97.5%), compared with PREDICT Day 1 (< 4) and Day 5 (< 2) (94.3% and 96.6% respectively).

Receiver operating curves comparing performance of each risk score in predicting IE in patients with SABScore Proportion of cohort Sensitivity (%) Specificity (%) Positive predictive value (%) Negative predictive value (%) PREDICT Day 1 ≥4 4/634 (0.6%) 0.0 99.3 0.0 94.3 PREDICT Day 5 ≥2 192/634 (30.2%) 58.3 71.4 10.9 96.6 VIRSTA ≥3 149/634 (23.5%) 66.7 79.1 16.1 97.5

**Figure:**
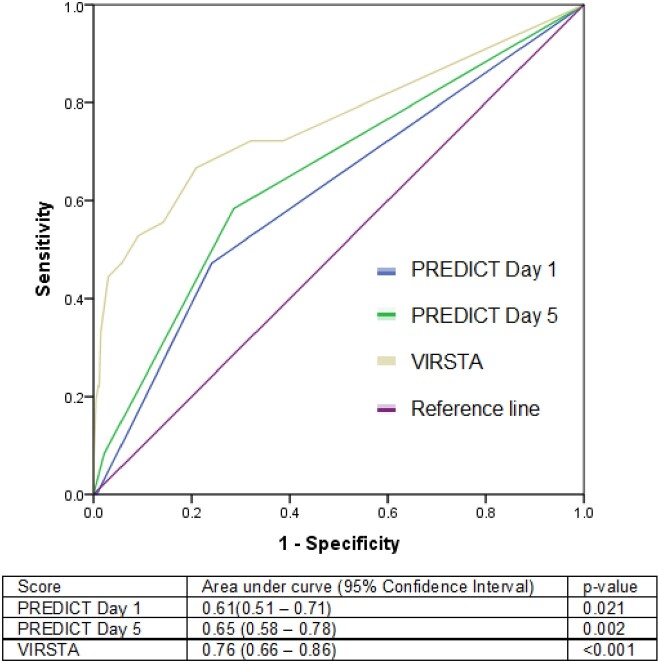

Comparing performance of each risk score at specified cut-offs in predicting IE in patients with SAB

**Figure:**
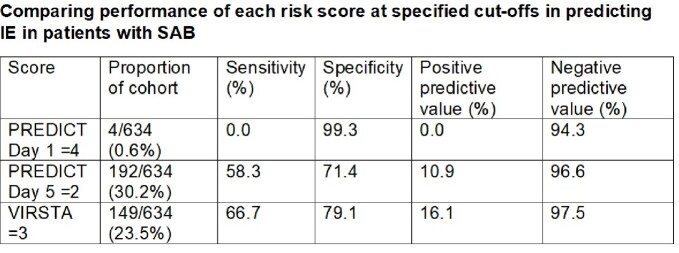

**Conclusion:**

Overall, the risk scores performed well in our Asian cohort. If applied, 23.5% of the cohort with a VIRSTA ≥ 3 required TEE, and a score of < 3 would have a negative predictive value of 97.5%.

**Disclosures:**

**All Authors**: No reported disclosures

